# Cytotoxicity and bioactivity of CIMMO DTA restorative cement in human activated-dental pulp cells *in vitro*

**DOI:** 10.1590/0103-644020256402

**Published:** 2025-04-14

**Authors:** Iandara de Lima Scardini, Karollyne dos Santos Spigariol, Marcelo dos Santos, Carla Renata Sipert, Letícia Martins Santos

**Affiliations:** 1 Department of Restorative Dentistry, School of Dentistry, University of São Paulo, São Paulo, Brazil.; 2 Department of Biomaterials and Oral Biology, School of Dentistry, University of São Paulo, São Paulo, Brazil.

**Keywords:** Bioceramic Restorative Cement, Cytotoxicity, Differentiation Potential Human Dental Pulp Cells.

## Abstract

Bioceramic materials have been introduced in dentistry due to their physical, chemical, and biological properties, which are considered advanced in clinical practice. According to the manufacturer, CIMMO DTA bioceramic restorative cement is a biological bioceramic cement and was introduced recently at a low cost compared to other bioceramic materials. However, no studies evaluated the effect of CIMMO DTA on human dental pulp cells (HDPC) activated with lipopolysaccharide (LPS). Therefore, this study aimed to evaluate the cytotoxicity and the osteo/odontogenic differentiation potential in HDPC activated with LPS exposed to CIMMO DTA compared to Hydro-C. HDPC activated or not with LPS were cultured, plated, and exposed to different dilutions of CIMMO DTA and Hydro-C extracts. At 24h, 48h, and 72h, the cells were subjected to the MTT assay. The osteo/odontogenic mineralization potential of cells was evaluated through the Alizarin red S staining assay at a 21-day time point. Statistical analysis was performed using a two-way analysis of variance (ANOVA) followed by Tukey's post-test (p<0.05). In general, the tested materials unaffected the metabolic activity and viability of cells in all dilutions evaluated. The CIMMO DTA reduced the osteo/odontogenic differentiation potential of the HDPC according to qualitative and quantitative results at dilutions of 1:8. Therefore, the results of the present study suggested that CIMMO DTA presents limited bioactive potential on HDPC in vitro.

## Resumo

Os materiais biocerâmicos têm sido introduzidos na Odontologia devido às propriedades físicas, químicas e biológicas consideradas avançadas na prática clínica. Segundo o fabricante, o cimento restaurador biocerâmico CIMMO DTA é um cimento biocerâmico biológico e foi introduzido recentemente com baixo custo em comparação com outros materiais biocerâmicos. No entanto, nenhum estudo avaliou o efeito do CIMMO DTA nas células da polpa dentária humana (HDPC) ativadas com lipopolissacarídeo (LPS). Portanto, este estudo teve como objetivo avaliar a citotoxicidade e o potencial de diferenciação osteo/odontogênica em HDPC ativadas com LPS exposto ao CIMMO DTA em comparação ao Hydro-C. HDPC ativadas ou não com LPS foram cultivadas, plaqueadas e expostas a diferentes diluições dos extratos CIMMO DTA e Hydro-C. Às 24h, 48h e 72h, as células foram submetidas ao ensaio MTT. O potencial de mineralização osteo/odontogênica das células foi avaliado através do ensaio de coloração Alizarin red S em um período de 21 dias. A análise estatística foi realizada por meio de análise de variância a dois critérios (two-way ANOVA) seguida de pós-teste de Tukey (p<0,05). Em geral, os materiais testados não afetaram a atividade metabólica e a viabilidade das células em todas as diluições avaliadas. O CIMMO DTA reduziu o potencial de diferenciação osteo/odontogênica das HDPC conforme resultados qualitativos e quantitativos em diluições de 1:8. Portanto, os resultados dos presentes estudos sugerem que o CIMMO DTA apresenta potencial bioativo limitado em HDPC in vitro.

**Figure:**
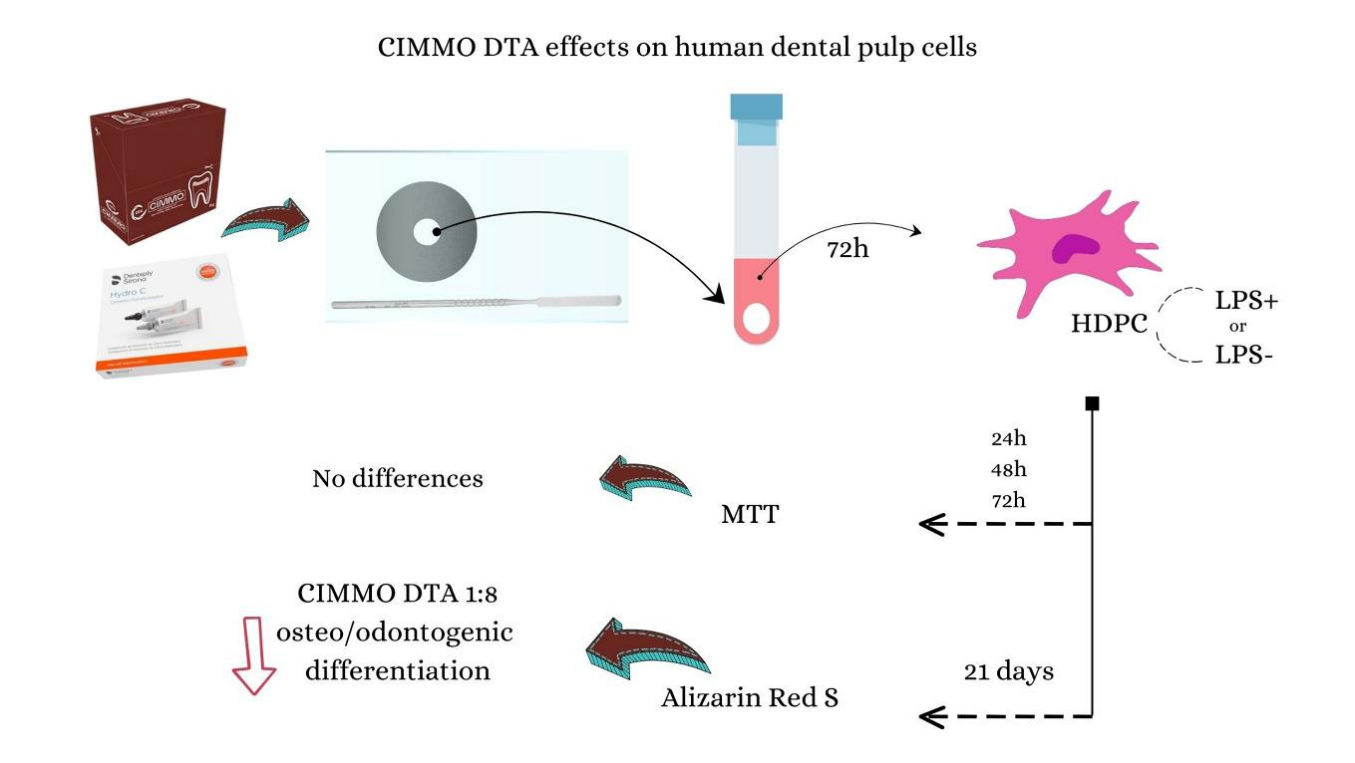

## Introduction

It is an international consensus that restorative interventions are needed for cavitated carious lesions that are difficult for the patient to clean, which may lead to further progression or those with significant tooth structure loss that cannot be simply sealed ^1^. The procedure aims to aid plaque control and thereby manage caries activity; restore the tooth's function, form, and aesthetics; and protect the pulp-dentine complex ^2^. The repair capacity of the pulp tissue is fundamental for tooth longevity, and preserving pulpal health should be prioritized for deeper lesions in teeth with vital pulp. The capping or base/liner agents have traditionally been used in these cases. They must preserve the pulp tissue vitality, promote cell survival and proliferation, be biologically neutral, and stimulate the formation of repair dentin ^3^ ^,^ ^4^ ^,^ ^5^.

In the last decades, bioceramic materials have been introduced in Dentistry, and due to their physical, chemical, and biological properties, are considered an important advance in clinical practice ^6^. Among the various clinical indications, some of these materials have been used as alternatives for pulp capping, and studies showed promising success rates ^7^ ^,^ ^8^ ^,^ ^9^ due to excellent biocompatibility and antimicrobial activity, adequate seal, and bioactivity showed by the capacity of induction of mineralized tissue ^7^ ^,^ ^8^ ^,^ ^9^ ^,^ ^10^. According to Abdul et al. ^9^, some bioceramic materials have been demonstrated to be promising and could be considered the future of capping materials.

According to the interaction with surrounding tissues, bioceramic materials can be classified as bioinert, bioactive, or biodegradable ^6^ ^,^ ^11^. The property of having a biological effect or a biological action denotes that a restorative material is bioactive ^12^. CIMMO DTA bioceramic restorative material (CIMMO, Pouso Alegre, MG) was recently introduced. According to the manufacturer, it is a biological bioceramic cement composed of mineral oxides in the form of fine hydrophilic particles suitable for restorations as a base or liner, which induces remineralization (CIMMO, 2023). Compared to other bioceramic restorative materials, CIMMO DTA is cost-effective.

In cases of traumatic or bacterial injury, pulp tissue is often exposed to biomaterials in an inflammatory state. Lipopolysaccharide (LPS), a well-known virulence factor, triggers inflammatory responses by inducing the release of pro-inflammatory cytokines and modulating cellular activity ^13^. Activating human dental pulp cells (HDPC) with LPS in vitro simulates an inflamed microenvironment, closely mimicking the clinical conditions of pulp inflammation. To date, no studies have evaluated the effect of CIMMO DTA on HDPC activated with LPS in vitro. Therefore, the present study aimed to evaluate the 1) cytotoxicity of CIMMO DTA in HDPC activated with LPS and 2) osteo/odontogenic differentiation potential of HDPC activated with LPS and exposed to CIMMO DTA compared to Hydro-C (Dentsply Sirona, Charlotte, EUA).

## Materials and methods

### Cell Culture

This study was approved by the local Ethics Committee (Process # 6.104.012). Human dental pulp cells (HDPC) were established by using the explant technique. The dental pulp was removed from a third molar, minced, and placed at culture flasks with low volumes of culture medium composed of alpha-minimum essential medium (α-MEM) (Sigma-Aldrich, St Louis, MO) with 10% FBS (Gibco Life Technologies, Grand Island, NY) and antibiotics (100 µg/mL penicillin, 100 µg/mL streptomycin, 0.5 mg/mL amphotericin B - Invitrogen) under standard culture conditions (37°C, 100% humidity, 5% CO_2_, and 95% air) until reaching 80% of confluence. Cells in the third passage were used for the experiments.

### Preparation of materials extracts

CIMMO DTA and Hydro-C compositions according to manufacture information are presented in Table 1. The materials were manipulated according to the manufacturer's instructions. For Hydro-C, equal volumes of base and catalyst (1:1) were mixed for a maximum of 10 seconds, while for CIMMO DTA, a scoop of material was divided into two portions, and separate drops of liquid were mixed with the powder using a small flexible spatula until the desired consistency (putty) was achieved. Each mixture was placed into a round metal device designed for the production of discs (5 x 3 mm) during setting time. Discs were removed and placed in a tube containing 1mL of incomplete alpha-minimum essential medium (α-MEM) (Sigma-Aldrich, St Louis, MO). After 72h of incubation under standard conditions (37°C, 100% humidity, 5% CO2, and 95% air), the extracts were filtered by 0.22-µm pore size membranes (Millipore) and freezed at -80ºC.

**Table 1:** CIMMO DTA and Hydro-C compositions and lot number

Material	Composition	Lot number
CIMMO DTA	Calcium Oxide, Calcium Carbonate, Magnesium Oxide, Dicalcium Silicate, Aluminum Oxide, Sodium Oxide, Potassium Oxide and Pozzolone	009
Hydro-C	Base past: Glycol Salicylate Ester, Calcium Phosphate, Calcium Tungstate, Zinc Oxide and Mineral Dyes.	
	Catalyst past: Ethyltoluene Sulfonamide, Calcium Hydroxide, Zinc Oxide, Titanium Dioxide, Zinc Stearate, and Dyes Minerals	3813240

### Cytotoxicity Assay

The cytotoxicity of Cimmo DTA and Hydro-C extracts, at dilutions of 1:4 and 1:8, was assessed in human dental pulp cells (HDPC) using the 3-(4,5-Dimethylthiazol-2-yl)-2,5-diphenyltetrazolium bromide (MTT) assay, following the guidelines outlined in the ISO 10993-12:2012(E). HDPC were seeded in triplicate at a density of 1.25 x 10^4^ cells per well in 96-well plates. Part of the cells were pre-activated with Escherichia coli lipopolysaccharide (LPS; 1μg/mL) (Sigma-Aldrich) for seven days previous to plate distribution. Following a 24-hour incubation period, the medium was replaced with α-minimum essential medium (α-MEM) (Sigma-Aldrich, St Louis, MO) with 1% FBS (Gibco Life Technologies, Grand Island, NY) to allow cell adaptation. Subsequently, cells were exposed to the Cimmo DTA and Hydro-C extracts diluted 1:4 or 1:8 in α-MEM for 24, 48, and 72 hours. Cells with α-MEM only (Sigma-Aldrich, St Louis, MO) represented the negative control group. At the respective time points, the cell supernatant was replaced with 20 µL of an MTT solution (5 mg/mL) (Sigma-Aldrich, St. Louis, MO, USA) in phosphate-buffered saline (PBS), followed by 180 µL of α-MEM 10% FBS. The cells were then incubated at 37°C for 4 hours, shielded from light. After incubation, the MTT solution was aspirated and replaced with 100 µL of dimethyl sulfoxide (DMSO) (Synth, Diadema, SP, Brazil). Optical density was measured at 570 nm using a Synergy HT microplate reader (Biotek Instruments, Inc., Winooski, VT, USA).

### Alizarin red S staining

The interference of Cimmo DTA and Hydro-C extracts (diluted 1:8 and 1:16) in the osteo/odontogenic mineralization potential of HDPC was evaluated using an Alizarin red S staining assay. Once pre-activated with lipopolysaccharide (LPS) for seven days or left untreated, cells were seeded in sextuplicate at a density of 1.25 x 10^4^ cells per well in 96-well plates and kept in either a proliferation medium (PM) or osteogenic induction medium (differentiation medium - DM) (proliferation medium supplemented with 2mmol/L KH_2_PO_4_ and 100 nmol dexamethasone) for 21 days. Following incubation, cells were fixed with 4% paraformaldehyde for 30 minutes, washed with PBS, and stained with 40 mmol/L Alizarin red S solution (Cat. A5533, Sigma-Aldrich; pH = 4.2) for an additional 30 minutes. Macroscopic and microscopic (Nikon Eclipse Ti light microscope; 10x magnification) observations were conducted for each group, and semi-analytical densitometry analysis was performed to quantify calcium deposits by adding 10% ammonium hydroxide solution, followed by measurement of absorbance at 405 nm.

### Statistical analysis

Statistical analyses were conducted with GraphPad Prism 9.0 (GraphPad Software, San Diego, CA, USA). Normality was assessed using the Shapiro-Wilk test, followed by a two-way analysis of variance (ANOVA) and Tukey's post-test for multiple comparisons. Statistical significance was defined as p<0.05.

## Results

### Cytotoxicity

**Figure 1:**
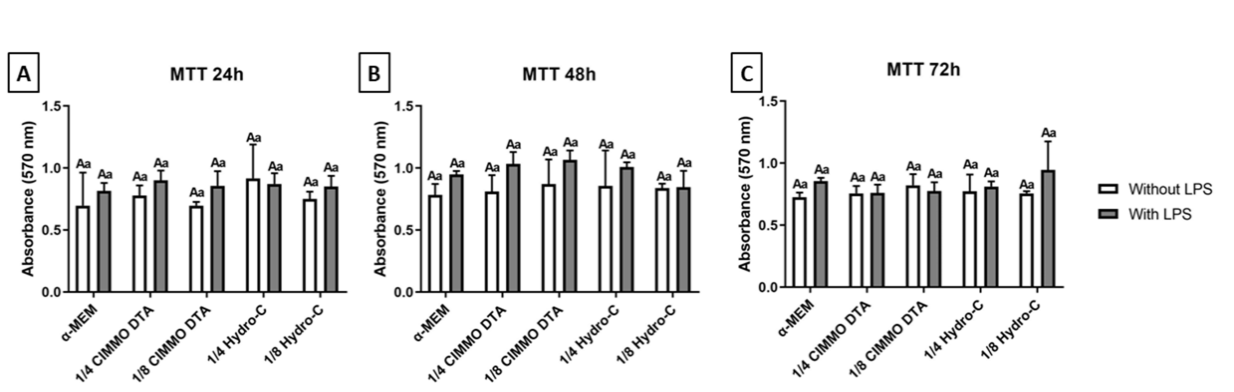
Absorbance (570nm) data obtained from the MTT assay after 24h (A), 48h (B), and 72h (C) of exposure to CIMMO DTA bioceramic cement extract and Hydro-C cement extract (diluted 1:4 and 1:8) in LPS-activated (1 ug/ml) or not activated human dental pulp cells. The results showed the mean and standard deviation of the experiments performed in triplicate. Different capital letters represent statistical differences between groups with the same stimulus. Different lowercase letters represent statistical differences among groups of different stimuli (Two-Way ANOVA with Tukey test, p <0.05).

The results of the MTT assay (Figure 1) indicate that the dilutions of the tested materials did not affect both metabolic activity and HDPC viability, irrespective of the duration of exposure (24, 48, or 72 hours). According to the ISO 10993-5:1999 (E) guidelines, biomaterials, such as CIMMO DTA, are classified as cytotoxic when cell viability is reduced by more than 30%. In this study, CIMMO DTA did not exhibit cytotoxic effects under the experimental conditions. Additionally, interaction analysis between the materials and LPS did not yield statistically significant findings (Figure 1A, B, and C) (p>0.05).

### Osteo/odontogenic differentiation potential

**Figure 2:**
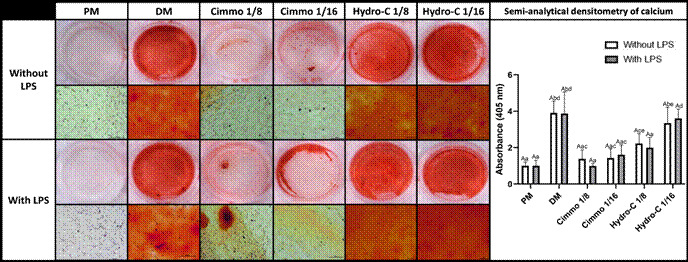
Mineralization potential of human dental pulp cells (HDPC) after 21 days of exposure to two dilutions (1:8 and 1:16) of Cimmo DTA and Hydro-C extracts, with or without lipopolysaccharide (LPS) stimulation (1 µg/mL) detected by alizarin red S staining. The figure presents both macroscopic and microscopic views of the cells, with and without LPS activation (calibration bars: 100µm). Additionally, the graph depicts the absorbance measurements (405 nm) obtained from the quantification of alizarin staining through elution. The results are presented as the mean and standard deviation of experiments conducted in sextuplicate. Different capital letters represented statistical differences in groups with the same stimulus. Different lowercase letters represented statistical differences in different stimulus groups (Two-Way ANOVA with Tukey test, p <0,05).

Despite not exhibiting cytotoxicity towards HDPC, CIMMO DTA at dilutions of 1:8 and 1:16 resulted in significantly reduced calcium deposition compared to the positive control (DM), as confirmed both qualitatively and quantitatively through semi-analytical calcium densitometry (Figure 2). In contrast, Hydro-C demonstrated a cellular response similar to the positive control group (DM) qualitatively. Nevertheless, quantitative analysis revealed a statistically significant reduction in calcium formation at the higher tested dilution of Hydro-C (1:8) compared to the positive control (DM) (Figure 2). Interestingly, the presence of LPS did not alter the osteo/odontogenic differentiation potential regardless of the material or dilution tested (Figure 2).

## Discussion

During restorative procedures, the use of protective materials, like liners and bases, aims to preserve pulp health and stimulate tertiary dentine layer deposition ^5^. The present study showed that considering cell viability, both CIMMO DTA and Hydro-C presented similar cytotoxicity in vitro, after being diluted at least four times (Fig. 1). On the other hand, they differently affected osteo/odontoblast differentiation of HDPC depending on eluates dilution. Both phenomena were not affected by LPS activation (Fig. 2).

Bioceramics comprise a group of biomaterials classified as bioinert, bioactive, or biodegradable according to their interaction with surrounding tissues ^6^ ^,^ ^11^. Calcium silicate-based materials are a small subset of bioceramics and are primarily classified as bioactive due to their ability to interact with the surrounding tissues, inducing a mineral tissue deposition and having the potential to heal biological tissues ^6^ ^,^ ^11^. Calcium silicate-based materials can be used as dental pulp protective materials since they have shown promise as the future of capping materials ^7^ ^,^ ^8^ ^,^ ^9^. Previous studies showed a significant potential to induce mineralization of these materials in vitro ^14^ ^,^ ^15^. Furthermore, a clinical trial compared the thickness of dentin formed after indirect pulp capping using calcium-hydroxide cement and MTA in primary teeth. The results showed better dentin formation with MTA ^16^.

The composition of CIMMO DTA presents mineral oxides, dicalcium silicate, and pozzolan. According to the manufacturer, pozzolan is associated with the formation of calcite granules, which is responsible for remineralization. Kim et al. ^17^ describe pozzolan as a type of siliceous material that reacts chemically with Ca(OH)_2_ to form compounds with cement-like properties. A previous study evaluated the biological properties of pozzolan-based pulp capping material, demonstrating excellent biocompatibility besides tertiary dentin induction, both in vitro and in vivo ^18^.

Calcium-hydroxide cement has long been considered the gold standard for protecting pulp tissue from irritations caused by restorative procedures ^5^ ^,^ ^9^ ^,^ ^19^. Despite its widespread use, it has several disadvantages, including high solubility and the induction of a porous dentin layer ^9^ ^,^ ^19^. Additionally, previous studies have demonstrated that calcium-hydroxide cement exhibits higher cytotoxicity than calcium silicate-based materials ^15^ ^,^ ^20^.

In the present study, we evaluated the cytotoxicity of CIMMO DTA and Hydro-C through the MTT assay. Our results showed no statistically significant differences in cytotoxicity among all evaluated dilutions of CIMMO DTA, Hydro-C, and the control group (culture medium only). Additionally, no significant increase in metabolic activity was observed with the tested materials, indicating that CIMMO DTA and Hydro-C did not promote cell proliferation. This suggests that CIMMO DTA and Hydro-C did not affect HDPC viability or proliferation under the conditions tested. However, it is important to note that the MTT assay measures mitochondrial activity, and other parameters of cell cytotoxicity could provide complementary insights into our findings.

Due to the dentinal permeability in teeth with deep carious, pulpal inflammation is usually observed during restorative procedure execution ^21^. Dental pulp cells typically proliferate, migrate, and differentiate into odontoblast-like cells, in turn forming tertiary dentin during pulp wound healing ^15^ ^,^ ^21^ ^,^ ^22^. According to the manufacturer, CIMMO DTA is a dentinal substitute that stimulates dentin layer formation, suggesting bioactivity. In our study, even the high concentration of CIMMO DTA (1:4) did not affect cell viability, so we proceeded to test lower concentrations (1:8 and 1:16) to evaluate their potential impact on osteo/odontoblast differentiation. Our results showed that exposing HDPC, whether activated with LPS or not, to CIMMO DTA inhibited osteo/odontoblast differentiation at both evaluated dilutions (p<0.05). Similarly, Hydro-C at a 1:8 dilution also inhibited differentiation (Fig. 2). However, at a 1:16 dilution, Hydro-C stimulated osteo/odontoblast differentiation comparable to the positive control (DM) (P>0.05).

Compared to findings in the literature that demonstrate the bioactive potential of other bioceramic materials, our study suggests that CIMMO DTA has limited bioactive potential. We were unable to directly connect the properties of CIMMO DTA's components to the observed differentiation inhibition. In contrast, the literature indicates that dicalcium silicate microparticles can modulate the expression of circRNAs and mRNAs in BMSC to promote osteogenesis ^23^; different mineral oxides nanoparticles, such as zinc and magnesium, could upregulate osteogenic cell differentiation ^24^ ^,^ ^25^; and pozzolan is related to calcite granule formation, which is responsible for remineralization, as previously described.

## Conclusion

Prior to our investigation, no studies had assessed the biological properties of CIMMO DTA. Although in vitro studies have inherent limitations, our research provided valuable insights into the responses of HDPC concerning cytotoxicity and osteo/dental differentiation potential when exposed to CIMMO DTA. Future analyses examining cellular responses in vitro can further enhance our dataset. Additionally, considering the experimental conditions of this study, conducting an in vivo investigation can offer complementary insights into the biological properties of CIMMO DTA.

## References

[1] Schwendicke F, Frencken JE, Bjørndal L, Maltz M, Manton DJ, Ricketts D (2016). Managing Carious Lesions: Consensus Recommendations on Carious Tissue Removal. Adv Dent Res.

[2] Kidd EAM (2004). How ‘Clean’ Must a Cavity Be before Restoration?. Caries Res.

[3] Motwani N, Ikhar A, Nikhade P, Chandak M, Rathi S, Dugar M (2021). Premixed bioceramics: A novel pulp capping agent. J Conserv Dent.

[4] Murray P, About I, Lumley P, Franquin JC, Remusat M, Smith A (2000). Human odontoblast cell numbers after dental injury. J Dent.

[5] Taghvaei N, Ghavami-Lahiji M, Evazalipour M, Tayefeh Davalloo R, Zamani E (2023). Ion release, biocompatibility, and bioactivity of resin-modified calcium hydroxide cavity liners. BMC Oral Health.

[6] Donnermeyer D, Bürklein S, Dammaschke T, Schäfer E (2019). Endodontic sealers based on calcium silicates: a systematic review. Odontology.

[7] Suhag K, Duhan J, Tewari S, Sangwan P (2019). Success of Direct Pulp Capping Using Mineral Trioxide Aggregate and Calcium Hydroxide in Mature Permanent Molars with Pulps Exposed during Carious Tissue Removal: 1-year Follow-up. J Endod.

[8] Linu S, Lekshmi MS, Varunkumar VS, Sam Joseph VG (2017). Treatment Outcome Following Direct Pulp Capping Using Bioceramic Materials in Mature Permanent Teeth with Carious Exposure: A Pilot Retrospective Study. J Endod.

[9] Abdul MSM, Murali N, Rai P, Mirza MB, Salim S, Aparna M (2021). Clinico-Histological Evaluation of Dentino-Pulpal Complex of Direct Pulp Capping Agents: A Clinical Study. J Pharm Bioallied Sci.

[10] Elshamy FM, Elraih H, Gupta I, Idris FA (2016). Antibacterial Effect of New Bioceramic Pulp Capping Material on the Main Cariogenic Bacteria. J Contemp Dent Pract.

[11] Surya Raghavendra S, Jadhav GR, Gathani KM, Kotadia P (2017). Bioceramics in Endodontics - A Review. J Istanbul Univ Fac Dent.

[12] Kunert M, Lukomska-Szymanska M (2020). Bio-Inductive Materials in Direct and Indirect Pulp Capping-A Review Article. Materials.

[13] Pedrosa MS, Vilela HS, Rahhal JG, Bueno NP, Lima FS, Nogueira FN (2022). Exposure to lipopolysaccharide and calcium silicate-based materials affects the behavior of dental pulp cells. Braz Dent J.

[14] Gomes-Cornélio AL, Rodrigues EM, Salles LP, Mestieri LB, Faria G, Guerreiro-Tanomaru JM (2017). Bioactivity of MTA Plus, Biodentine and an experimental calcium silicate-based cement on human osteoblast-like cells. Int Endod J.

[15] Manaspon C, Jongwannasiri C, Chumprasert S, Sa-Ard-Iam N, Mahanonda R, Pavasant P (2021). Human dental pulp stem cell responses to different dental pulp capping materials. BMC Oral Health.

[16] George V, Janardhanan S, Varma B, Kumaran P, Xavier A (2015). Clinical and radiographic evaluation of indirect pulp treatment with MTA and calcium hydroxide in primary teeth (in-vivo study). J Indian Soc Pedod Prev Dent.

[17] Kim MA, Rosa V, Neelakantan P, Hwang YC, Min KS (2021). Characterization, Antimicrobial Effects, and Cytocompatibility of a Root Canal Sealer Produced by Pozzolan Reaction between Calcium Hydroxide and Silica. Materials.

[18] Park SJ, Heo SM, Hong SO, Hwang YC, Lee KW, Min KS (2014). Odontogenic Effect of a Fast-setting Pozzolan-based Pulp Capping Material. J Endod.

[19] Qureshi A (2014). Recent Advances in Pulp Capping Materials: An Overview. J Clin Diagn Res.

[20] Camargo SEA, Camargo CHR, Hiller KA, Rode SM, Schweikl H, Schmalz G (2009). Cytotoxicity and genotoxicity of pulp capping materials in two cell lines. Int Endod J.

[21] Ricucci D, Loghin S, Siqueira JF (2014). Correlation between clinical and histologic pulp diagnoses. J Endod.

[22] Goldberg M (2011). Pulp Healing and Regeneration. Adv Dent Res.

[23] Zhong W, Li X, Pathak JL, Chen L, Cao W, Zhu M (2020). Dicalcium silicate microparticles modulate the differential expression of circRNAs and mRNAs in BMSCs and promote osteogenesis via circ_1983-miR-6931-Gas7 interaction. Biomater Sci.

[24] Tang Y, Rajendran P, Veeraraghavan VP, Hussain S, Balakrishna JP, Chinnathambi A (2021). Osteogenic differentiation and mineralization potential of zinc oxide nanoparticles from Scutellaria baicalensis on human osteoblast-like MG-63 cells. Mater Sci Eng C.

[25] Chen Y, Sheng W, Lin J, Fang C, Deng J, Zhang P (2022). Magnesium Oxide Nanoparticle Coordinated Phosphate-Functionalized Chitosan Injectable Hydrogel for Osteogenesis and Angiogenesis in Bone Regeneration. ACS Appl Mater Interfaces.

